# Using Wearable Devices to Monitor Physical Activity in Patients Undergoing Aortic Valve Replacement: Protocol for a Prospective Observational Study

**DOI:** 10.2196/20072

**Published:** 2020-11-12

**Authors:** Giulia Lorenzoni, Danila Azzolina, Chiara Fraccaro, Alessandro Di Liberti, Augusto D'Onofrio, Chiara Cavalli, Tommaso Fabris, Gianpiero D'Amico, Giorgia Cibin, Luca Nai Fovino, Honoria Ocagli, Gino Gerosa, Giuseppe Tarantini, Dario Gregori

**Affiliations:** 1 Unit of Biostatistics, Epidemiology and Public Health Department of Cardiac, Thoracic, Vascular Sciences and Public Health University of Padova Padova Italy; 2 Interventional Cardiology Unit Department of Cardiac, Thoracic, Vascular Sciences and Public Health University of Padova Padova Italy; 3 Cardiac Surgery Unit Department of Cardiac, Thoracic, Vascular Sciences and Public Health University of Padova Padova Italy

**Keywords:** surgical aortic valve replacement, transcatheter aortic valve replacement, physical function, wearable devices

## Abstract

**Background:**

In last few decades, several tools have been developed to measure physical function objectively; however, their use has not been well established in clinical practice.

**Objective:**

This study aims to describe the preoperative physical function and to assess and compare 6-month postoperative changes in the physical function of patients undergoing treatment for aortic stenosis with either surgical aortic valve replacement (SAVR) or transcatheter aortic valve replacement (TAVR). The study also aims to evaluate the feasibility of wearable devices in assessing physical function in such patients.

**Methods:**

This is a prospective observational study. The enrollment will be conducted 1 month before patients’ SAVR/TAVR. Patients will be provided with the wearable device at baseline (activity tracker device, Garmin vívoactive 3). They will be trained in the use of the device, and they will be requested to wear it on the wrist of their preferred hand until 12 months after SAVR/TAVR. After baseline assessment, they will undergo 4 follow-up assessments at 1, 3, 6, and 12 months after SAVR/TAVR. At baseline and each follow-up, they will undergo a set of standard and validated tests to assess physical function, health-related quality of life, and sleep quality.

**Results:**

The ethics committee of Vicenza in Veneto Region in Italy approved the study (Protocol No. 943; January 4, 2019). As of October 2020, the enrollment of participants is ongoing.

**Conclusions:**

The use of the wearable devices for real-time monitoring of physical activity of patients undergoing aortic valve replacement is a promising opportunity for improving the clinical management and consequently, the health outcomes of such patients.

**Trial Registration:**

Clinicaltrials.gov NCT03843320; https://tinyurl.com/yyareu5y

**International Registered Report Identifier (IRRID):**

DERR1-10.2196/20072

## Introduction

High levels of physical activity are essential for the success of cardiac procedures; it has been established that ad hoc cardiac rehabilitation programs improve patients' functional recovery through exercise therapy [1]. Ad hoc exercises (both preoperative and postoperative) have been demonstrated to reduce the likelihood of postoperative complications (eg, postoperative pulmonary complications and thromboembolism), facilitate physical recovery, and reduce the length of hospital stay [2].

Aortic stenosis is the most common valvular disease in patients aged over 75 years with a prevalence of about 3%, and 1 out of 8 of such patients is affected by moderate/severe disease [3]. For a long time, surgical aortic valve replacement (SAVR) has been the standard of care for treatment of aortic stenosis treatment. However, transcatheter aortic valve replacement (TAVR) has recently emerged as an alternative for the treatment of aortic stenosis in selected patients [4-6], which seems to result in a faster physical recovery compared to SAVR. However, most of the studies on patients receiving SAVR and TAVR have focused on postoperative changes in health-related quality of life (HRQoL). The physical function has been assessed as a parameter of HRQoL (eg, 36-Item Short Form Health Survey [SF-36]), showing that patients receiving TAVR generally show more significant improvements in HRQoL at 3-, 6-, and 9-month follow-ups [7-9] than those in patients receiving SAVR [10]. A few studies have explicitly concentrated on the assessment of physical function. It has been shown that the postoperative changes in physical function of patients undergoing valve replacement are mainly affected by the severity of their condition [11], and physical function in such patients can be improved through ad hoc rehabilitation programs [12-14].

It is worth pointing out that there is a lack of specific data on the trajectories of physical recovery in patients receiving SAVR and TAVR, despite their widely acknowledged key roles in affecting postoperative outcomes, especially among elderly who are more prone to develop postoperative complications. Given the close relationship of physical activity with the outcomes of cardiac procedures, there is a growing interest in improving assessment of physical activity. Undoubtedly, several methods are available to assess physical function (and are widely used in both everyday clinical practice and clinical research) [15]. However, such approaches present several limitations, and the main one is that physical function is self-reported (and self-rated) by the patient. Self-reporting can be biased (eg, recall and desirability biases), and this might pose a barrier to further improvement in patients' recovery from surgery. In the last few decades, several tools have been developed to measure physical function objectively. Particularly, commercially available wearable technologies are increasingly used for both collecting and promoting patients' activity [16] in the health care setting. A recent review in the field has shown a broad spectrum of applications of such technologies for several purposes, including health promotion, health maintenance, and clinical monitoring of patients with various pathological conditions or patients undergoing surgery [17]. The fact that such devices allow for continuous data collection presents a promising opportunity to improve the monitoring of patients, especially those with chronic diseases, allowing for early detection of changes in patients' activity that need to be investigated by the clinicians [18]. However, the use of such devices has not been well established in everyday clinical practice.

This study aims to describe baseline (preoperative) physical function and to assess and compare 6-month postoperative changes in the physical function of patients undergoing treatment for aortic stenosis with either SAVR or TAVR with Edwards valve implants. It also aims to evaluate the feasibility of wearable devices in assessing physical function in such patients.

## Methods

This is a prospective observational study.

### Study Population

The study will enroll patients undergoing SAVR and TAVR. Aortic valve replacement interventions will be mandated either by patients’ symptoms or by indications proposed by the current guidelines and approved by the local heart team.

The inclusion criteria are as follows: (1) age, 75-90 years; (2) severe native aortic valve stenosis symptomatic for heart failure or angina; (3) indication to isolated TAVR or SAVR, approved by the heart team; (4) TAVR through the transfemoral approach; (5) SAVR by any access; (6) implantation of an Edwards valve (Sapien 3 and Sapien XT for TAVR; Inspiris Resilia, Edwards Intuity, and Carpentier-Edwards Perimount Magna Ease for SAVR); (7) not using walking aids; and (8) written informed consent. Patients not fitting the inclusion criteria, those with reduced life expectancy due to severe comorbidities (<1 year), and those with Parkinson disease will be excluded from the study.

### Study Procedures and Data Collection

**Figure 1:**
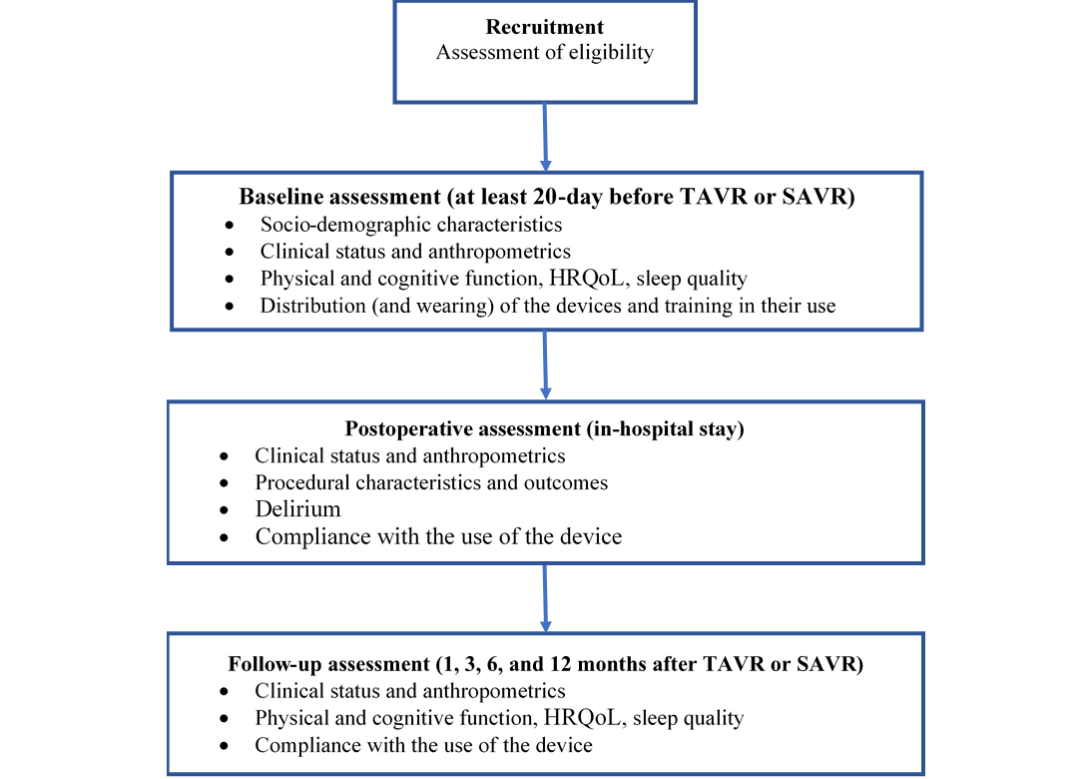
Flowchart of the study protocol. HRQoL: health-related quality of life; SAVR: surgical aortic valve replacement; TAVR: transcatheter aortic valve replacement.

Study procedures are reported in Figure 1. The enrollment will be performed 1 month before SAVR/TAVR. Patients will be provided with the wearable device at baseline. They will be trained in the use of the device, and they will be requested to wear it on the wrist of their preferred hand until 12 months after their SAVR/TAVR. After the baseline assessment, they will undergo 4 follow-up assessments at 1, 3, 6, and 12 months after their SAVR/TAVR.

At baseline and at each follow-up, they will undergo a set of standard and validated tests to assess their physical function, namely, 6-Minute Walk Test (6MWT), Duke Activity Status Index (DASI), Barthel Activities of Daily Living Index, and Instrumental Activity of Daily Living (IADL). Cognitive function will be assessed by Mini Mental State Examination (MMSE). Health-related quality of life will be assessed by SF-36 and Toronto Aortic Stenosis Quality of Life Questionnaire (TASQ). Sleep quality will be assessed by Epworth Sleepiness Scale (ESS).

### Study Device

Smartwatch activity tracker devices (vívoactive 3, Garmin) will be used in the study. Devices will be provided to the patients at the time of baseline assessment, along with a Bluetooth-paired smartphone with prepaid data-only SIM card and user interface customized for the study.

### Device Data Collection

Data will be collected from the wearable devices employing 2 complementary strategies to access both standard data (made available by Garmin and represented by daily activity statistics obtained through proprietary algorithms) and raw data.

1. Standard data will be collected using the Garmin Health API (application program interface). The standard data will be collected through the Garmin-Connect app installed on the smartphone. Patients will be requested to synchronize the device with their smartphone at least 3 times a week to allow the collection of the data stored in the device.
2. Raw data will be collected using an ad hoc app (developed for the study) powered by Garmin-Connect API. The data stored in the device will be automatically collected every time the phone and the device are close enough to be connected via Bluetooth (less than 8 meters). Patients will be instructed to keep the phone close to bed to allow data collection during nighttime. Once downloaded, the data will be stored in flexible and interoperable data transfer (FIT) files.

### Data Management

Site personnel trained in using the device will be available on workdays from 9 AM to 5 PM for technical assistance (by phone) about connection, synchronization, and setting of the smartphones and the devices. The same site personnel will send reminders (by phone) to patients if they do not regularly perform the data download for the standard data collection.

### Sample Size

The primary endpoint is represented by the potential gain—assessed through the 6MWT—in recovering physical function within the first 6 months after SAVR/TAVR. A sample size of 154 patients per group has been computed using a Kolmogorov-Smirnov test to evaluate the difference between the growth curves of 6MWT between the SAVR and TAVR groups. A simulation procedure has been performed assuming the following factors:

1. Logistic growth function for both groups.
2. Mean growth rate of 10% for both samples, assuming a mean time (useful to recover a carrying capacity of 270 meters) of 100 days for SAVR and 80 days for TAVR (with a mean reduction of recovery time consisting of 20 days).
3. A type I error probability equal to .05.
4. Each scenario consists of a combination of sample sizes from 100 to 200, and a difference between midpoint time recovery ranges from 5 to 30 days.

A statistical power of 0.9 has been reached with a difference between a recovery time of 20 days for 150 patients. For each scenario, 100 simulations have been performed by sampling *n* growth curves (1 for each patient), assuming the following growth function: 
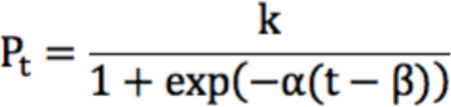


In this equation, α∼N (0.1,0.01), where α is the growth rate specifying the width of the sigmoidal curve; k is the carrying capacity; and β specifies the time when the curve reaches the midpoint of the growth trajectory.

**Figure 2:**
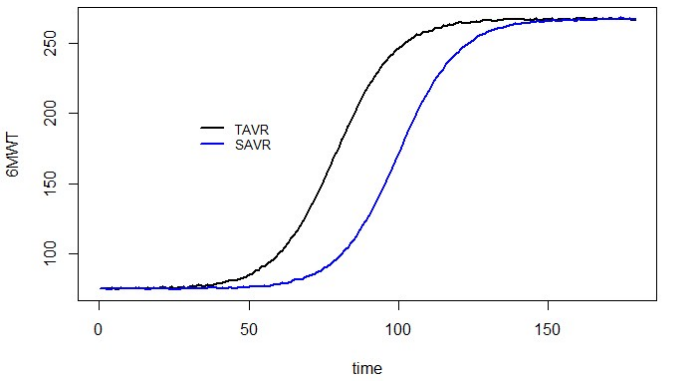
Simulated growth rate for the 6-Minute Walk Test (6MWT). SAVR: surgical aortic valve replacement; TAVR: transcatheter aortic valve replacement.

The curves have been pooled computing the mean within the sample, obtaining a mean SAVR and TAVR growth rate, as shown in Figure 2. Total patients to be enrolled are 340 (170 in each of the SAVR and TAVR groups), considering a dropout rate of 10%.

### Statistical Analysis

Data will be reported as median (interquartile range) values for continuous variables and as percentage (absolute number) values for qualitative variables. A Wilcoxon-Kruskal-Wallis test will be performed for continuous variables and Pearson chi-square tests for categorical ones.

A propensity score estimation will be provided to balance data in the SAVR and TAVR groups according to the baseline characteristics. A random forest classification algorithm [19] will be employed for propensity score computation.

A genetic algorithm [20] will be considered to match the data, taking into account the similarities in the estimated propensity scores. The algorithm automatically finds the set of matches, which minimizes the discrepancies between groups.

Once the data are matched, treatment groups will be compared to assess the quality of the matching procedure according to the baseline characteristics, reporting the same statistics as previously considered to describe patients receiving SAVR and TAVR.

Finally, a generalized estimating equations model [21] will be estimated on matched data to determine the time effect on the 6MWT performance in both treatment groups; the model will also include other confounding factors potentially affecting 6MWT.

### Validity of the Device in Measuring Physical Function

A validation study (ie, device vs observer in measuring the primary endpoint) will be performed during the 6MWT at each follow-up (1, 3, 6, and 12 months) and will involve all the patients enrolled in the study.

The number of steps performed during each 6MWT will be assessed both by the device and manually (using a step counter) by an independent observer. The manual counting of steps will be considered the gold standard. The number of steps recorded by the device will be compared to those counted manually (the gold standard).

The mean absolute percent error (MAPE) of the number of steps, as the average of the unsigned percentage error, will be computed to assess the agreement between the 2 methods, reporting at 95% confidence interval. A MAPE of less than 5% will be considered excellent, while a MAPE greater than 10% will be considered poor [22]. The validation of Garmin devices will also be performed and reported using Bland-Altman plots [23].

Moreover, the intraclass correlation coefficient (ICC) will be calculated for the number of steps measured manually and that obtained by the Garmin device. An ICC of ≥0.75 will be identified as excellent, 0.65-0.74 as good, 0.40-0.64 as fair, and <0.40 as poor [24].

The concordance between the number of steps recorded by the Garmin device and that by the gold standard (manual counting) will be evaluated at 1, 3, 6, and 12 months after SAVR or TAVR. Considering the multiplicity issues related to measures performed repeatedly, *P* value adjustment will be performed to control the inflation of the type I error rate of the experiment using the Holm procedure [25].

### Ethics Approval and Consent To Participate

The study was approved by the ethics committee of the province of Vicenza in the Veneto Region of Italy (Protocol No. 943; January 4, 2019). Each patient or legally authorized representative must provide a written informed consent for the study procedures.

## Results

Patient enrollment is ongoing. A total of 20 participants have been enrolled as of October 2020.

## Discussion

Objective measurement of physical function presents a promising opportunity to improve the postoperative management of cardiac patients. Demonstrating the validity of wearable devices in monitoring physical recovery in patients undergoing cardiac procedures will provide new insights in the clinical management of such patients, allowing for continuous monitoring and real-time readjusting of patients' therapy. For this reason, the interest in wearable devices is rapidly growing [26]. Wearable devices have been employed to monitor both lifestyle habits (eg, physical activity, dietary habits, or sleep) and clinical parameters (eg, heart rate) in both healthy people and those with acute or chronic diseases. Recently, these devices have been also used in the field of surgical care, mainly in the context of orthopedic surgery [27,28]. However, data on their use in the context of cardiac procedures (and in general, cardiovascular diseases) are scant, despite growing interest.

There are still several limitations in the use of such devices; therefore, their use in everyday clinical practice is not yet well established. Main challenges in the use of these devices are the choice of the device (which should be validated on the population of interest); patients' (or their caregivers’) training in the use of the device at home (eg, charging the device or connecting with the Bluetooth); and management of the large quantity of data collected by the device, which requires specific skills.

Lately, several wearable devices have been introduced in the market. A recent review [29] identified 81 studies indexed in PubMed that used one of these wearable devices for purposes of validation or data collection in research projects. The review advised that a research study should choose a wearable device that is most frequently used in the particular sector/care setting. Accordingly, this study will be using the aforementioned Garmin devices. Although we exclude patients with Parkinson disease in this study, it should be noted that another recent study [30] has validated a Garmin device’s accuracy in tracking the physical activity of patients with Parkinson disease.

Using wearable devices for the real-time monitoring of lifestyle habits and clinical parameters of patients undergoing cardiac surgery can present a promising opportunity for improving the clinical management and consequently, the health outcomes of such patients. Furthermore, information gained from this study can be helpful in providing patients contemplating intervention for aortic stenosis with practical counseling on expected changes in their functional status months after surgery.

## Abbreviations

6MWT: 6-Minute Walk Test
API: application program interface
DASI: Duke Activity Status Index
ESS: Epworth Sleepiness Scale
FIT: flexible and interoperable data transfer
HRQoL: health-related quality of life
IADL: Instrumental Activity of Daily Living
ICC: intraclass correlation coefficient
MAPE: mean absolute percent error
MMSE: Mini Mental State Examination
SAVR: surgical aortic valve replacement
SF-36: 36-Item Short Form Health Survey
TASQ: Toronto Aortic Stenosis Quality of Life Questionnaire
TAVR: transcatheter aortic valve replacement
